# PD-1 Dynamically Regulates Inflammation and Development of Brain-Resident Memory CD8 T Cells During Persistent Viral Encephalitis

**DOI:** 10.3389/fimmu.2019.00783

**Published:** 2019-04-17

**Authors:** Shwetank, Elizabeth L. Frost, Taryn E. Mockus, Heather M. Ren, Mesut Toprak, Matthew D. Lauver, Colleen S. Netherby-Winslow, Ge Jin, Jennifer M. Cosby, Brian D. Evavold, Aron E. Lukacher

**Affiliations:** ^1^Department of Microbiology and Immunology, Penn State College of Medicine, Hershey, PA, United States; ^2^Immunology and Molecular Pathogenesis Graduate Program, Emory University, Atlanta, GA, United States; ^3^Section of Neuropathology, Yale School of Medicine, New Haven, CT, United States; ^4^Department of Pathology, Microbiology and Immunology, University of Utah, Salt Lake City, UT, United States

**Keywords:** viral encephalitis, tissue-resident memory, CD8 T cells, PD-1, PD-L1, mouse polyomavirus, neuroinflammation

## Abstract

Programmed cell death-1 (PD-1) receptor signaling dampens the functionality of T cells faced with repetitive antigenic stimulation from chronic infections or tumors. Using intracerebral (i.c.) inoculation with mouse polyomavirus (MuPyV), we have shown that CD8 T cells establish a PD-1^hi^, tissue-resident memory population in the brains (bT_RM_) of mice with a low-level persistent infection. In MuPyV encephalitis, PD-L1 was expressed on infiltrating myeloid cells, microglia and astrocytes, but not on oligodendrocytes. Engagement of PD-1 on anti-MuPyV CD8 T cells limited their effector activity. NanoString gene expression analysis showed that neuroinflammation was higher in PD-L1^−/−^ than wild type mice at day 8 post-infection, the peak of the MuPyV-specific CD8 response. During the persistent phase of infection, however, the absence of PD-1 signaling was found to be associated with a lower inflammatory response than in wild type mice. Genetic disruption and intracerebroventricular blockade of PD-1 signaling resulted in an increase in number of MuPyV-specific CD8 bT_RM_ and the fraction of these cells expressing CD103, the αE integrin commonly used to define tissue-resident T cells. However, PD-L1^−/−^ mice persistently infected with MuPyV showed impaired virus control upon i.c. re-infection with MuPyV. Collectively, these data reveal a temporal duality in PD-1-mediated regulation of MuPyV-associated neuroinflammation. PD-1 signaling limited the severity of neuroinflammation during acute infection but sustained a level of inflammation during persistent infection for maintaining control of virus re-infection.

## Introduction

The inhibitory receptor PD-1 plays a dominant role in T cell exhaustion, a state of progressive loss of T cell function resulting from repetitive antigen stimulation such as in chronic viral disease or tumor development (1). Extensive work using experimental models of chronic infection [lymphocytic choriomeningitis virus (LCMV)-clone 13, simian immunodeficiency virus (SIV)] as well as analysis of T cells from individuals infected with human immunodeficiency virus (HIV), hepatitis C virus, and hepatitis B virus, demonstrate that blockade of PD-1 signaling restores T cell functionality (2). Engagement of PD-1 by its ligands PD-L1 (CD274/B7-H1) or PD-L2 (CD273/B7DC) results in recruitment of SHP-phosphatases proximal to the T cell receptor (TCR). These phosphatases inactivate kinase cascades induced by TCR signaling and thereby inhibit downstream pathways required for cytokine production, proliferation, and cytotoxicity (3, 4). PD-L1 is expressed on a variety of cell types while the expression of PD-L2 is limited to antigen presenting cells (5–7). CNS infection with neurotropic coronavirus induced the expression of PD-L1 but not PD-L2 on glia (8). In addition, only PD-1:PD-L1 interactions are responsible for inhibiting CD8 T cell effector function in mouse cytomegalovirus (MCMV) CNS infection (9). PD-1 mediated T cell exhaustion is characterized by increased expression of the transcription factors Eomesodermin (Eomes) and B lymphocyte-induced maturation protein-1 (Blimp-1), co-expression of inhibitory receptors PD-1, Tim3, and 2B4 and diminished effector function [Interferon (IFN)-γ and degranulation] (2, 10).

Accumulating evidence challenges the concept that PD-1 expression is solely synonymous with T cell dysfunction and senescence (11–13). PD-1 regulates T cell mobility in tissues (14) and T cell survival (10, 15). Additionally, in chronic high-viremic infections, T cell dysfunction is not absolute as shown by the emergence of CD8 T cell epitope-escape HIV late in infection (16), and increased viral titers after depletion of CD8 T cells in chronic SIV infection (17, 18). Recent reports support the concept that CD8 T cell exhaustion is a bona fide state of differentiation adapted by T cells, which enables them to survive and retain functionality during persistent infection (19, 20). Reversal of T cell exhaustion by blockade of the PD-1:PD-L1 axis indicates that exhausted T cells span a spectrum of dysfunction with the least exhausted cells, characterized as Tim3^−^PD-1^int^TCF-1^hi^CXCR5^hi^ cells in LMCV infection (21, 22), being more susceptible to functional resurrection after checkpoint inhibitor blockade. PD-1 or PD-L1 antibody-mediated blockade has shown remarkable effectiveness for certain types of cancer (23). Expression of PD-1 has recently been reported in human lung- and brain-resident CD8 T cells, with PD-1 signaling proposed to limit inadvertent deployment of effector mechanisms (24). Tissue-resident memory CD8 T cells are an essential component of the first line of defense against re-infection in non-lymphoid tissues (24–26). Whether PD-1 regulates CD8 T cell activity and affects memory differentiation in non-lymphoid tissues is an open question. Here we asked, in CNS persistent infection, if PD-1 operates to dampen CD8 T cell effector activity and modulate neuroinflammation.

Mouse polyomavirus (MuPyV), a non-enveloped virus with a covalently closed circular ~5-kb double-stranded DNA genome, is the founding member of the family *Polyomaviridae*. MuPyV establishes a systemic low-level persistent infection in mice; however, whether the virus persists as a smoldering infection or cycles between latency and reactivation has yet to be experimentally ascertained. Inoculation i.c. with MuPyV gives rise to a stable population of virus-specific PD-1^+^ tissue-resident memory CD8 T cells in the brain (bT_RM_) (27, 28). Although the role of PD-1 in modulating T cell function has been extensively investigated for virus-specific lymphoid CD8 T cells in the setting of chronic viremia (29), only a few studies have examined PD-1's impact on T_RM_ responding to a persistent viral CNS infection (8, 9, 30). In humans, JC polyomavirus (JCPyV) causes several aggressive CNS diseases, the most common being the frequently fatal demyelinating disease progressive multifocal leukoencephalopathy (PML) (31). JCPγV-specific CD8 T cells in PML patients express PD-1 (32). MCMV, Theiler's murine encephalomyelitis virus (TMEV), and JHM mouse hepatitis virus (JHMV) CNS infections demonstrate that excessive CD8 T cell effector activity is immunopathologic (8, 9, 30, 33). Whether PD-1 regulates neuroinflammation and formation of CD8 T_RM_ in a persistent infection model is incompletely understood.

In this study, we show that PD-1 acts to inhibit the effector functions of virus-specific CD8 bT_RM_ during MuPyV encephalitis. NanoString inflammatory gene expression analysis of brains of wild type (WT) and PD-L1^−/−^ mice infected with MuPyV revealed that PD-1 signaling controls the inflammatory response during acute infection. In striking contrast, the absence of PD-1 signaling during persistent infection resulted in lower neuroinflammation than in WT mice. PD-1 was further found to affect the differentiation of virus-specific CD8 bT_RM_. Together, these findings reveal a complex, dynamic impact of PD-1 on neuroinflammation and CD8 bT_RM_ formation and activity during persistent viral encephalitis.

## Materials and Methods

### Mice and Virus Inoculation

C57BL/6NCr (WT) female mice purchased from the Frederick Cancer Research and Development Center of the National Cancer Institute (Frederick, MD) and B7-H1^−/−^ (PD-L1^−/−^) mice [generously provided by C.C. Bergmann (Lerner Research Institute, Cleveland, OH) with approval of L. Chen (Yale School of Medicine, New Haven, CT)] were housed in accordance with the guidelines of the Institutional Animal Care and Use Committees and the Department of Comparative Medicine at the Pennsylvania State University College of Medicine. The Pennsylvania State University College of Medicine Animal Resource Program is accredited by the Association for Assessment and Accreditation of Laboratory Animal Care International (AAALAC). The Pennsylvania State University College of Medicine has an Animal Welfare Assurance on file with the National Institutes of Health's Office of Laboratory Animal Welfare; the Assurance Number is A3045-01. TCR-I mice expressing a TCR transgene specific for the large T Ag (LT-Ag) 206–215 amino acids from the SV40 virus and the MuPyV mutant encoding this epitope have been previously described (34, 35). At 7-12 weeks of age, anesthetized mice were i.c. inoculated by injecting the right frontal lobe with 30 μl of 2 × 10^7^ plaque-forming units (PFU) of MuPyV strain A2 in DMEM 5% FBS, as previously described (27, 33, 36).

### Quantification of MuPyV Genomes and LT-Ag mRNA

TaqMan real-time PCR was performed in an ABI StepOnePlus thermocycler (Applied Biosciences) with 10 μg of template DNA purified from tissues using the Maxwell 16 Research Instrument (Promega, Madison, WI) according to the manufacturer's instructions. Primers and amplification parameters are previously described (37). To determine MuPyV LT-Ag mRNA copy numbers, total RNA from FACS-purified cells was insolated using TRIzol (Ambion, USA) and cDNA was prepared using RevertAid H minus reverse transcriptase (Thermo Scientific, Waltham, MA), as per manufacturer's instructions. Quantitative PCR (qPCR) was performed using FastStart Universal Probe Master (ROX) mix (Sigma-Aldrich) and an ABI PRISM 5700 sequence detection system (Applied Biosystems, Foster, CA), with protocol and primer set as previously described (38).

### Bone Marrow Dendritic Cell Culture

Bone marrow was flushed from the femurs and tibias of WT and PD-L1^−/−^ mice using a 30-gauge needle and syringe loaded with DMEM 10% FBS. Red blood cells were lysed with ACK buffer. Bone marrow-derived cells were plated (5 × 10^6^ cells/100 mm diameter Petri dish) and cultured in DMEM 10% FBS with GM-CSF (20 ng/ml) at 37°C, with the media changed every 3 days. After 10 days, differentiated bone marrow dendritic cells (BMDCs) were harvested by gentle trypsinization [trypsin-EDTA (0.25%) (ThermoFisher Scientific) for < 1 min] to release loosely adherent cells. BMDCs were re-plated (3 × 10^6^ cells/100 mm diameter Petri dish in DMEM 10% FBS) with 100 U/ml IFN-γ (PeproTech US, Rocky Hill, NJ) to induce antigen presentation and PD-L1 expression and were incubated overnight at 37°C. BMDCs were transferred to a 96-well plate (3 × 10^5^ cells/well) and incubated with 10 μM LT359 peptide at 37°C for ~6 h prior to co-culture with T cells isolated from brain or spleen.

### Cell Isolation, Intracellular Cytokine Staining, and Flow Cytometry

For isolation of neural and mononuclear cells (39), anesthetized mice were perfused transcardially with 30 ml heparinized PBS (100 U/ml). Brains were minced and digested with Collagenase I (100 mg/100 ml) for 15 min at 37°C. Single-step 37% Percoll centrifugation was used to remove myelin from brain homogenates. Cells were washed and incubated with antibodies against CD45 (1:200 dilution, 30-F11), CD11b (1:200 dilution, M1/70), GLAST (Miltenyi, Bergisch Gladbach, Germany, 1:200, ACSA-1), and O4 (product code 130-095-891, Miltenyi, Bergisch Gladbach, Germany; 1:20 dilution). For all other experiments, post perfusion, finely minced brains were digested with collagenase I (Worthington Biochemical, Lakewood, NJ) (40 mg/100 ml) for 20 min at 37°C, followed by a two-step 44%/66% Percoll gradient to remove myelin and cell debris. Spleen and brain cells were exposed to Fixable Viability Dye (eBioscience, San Diego, CA) and Fc Block (BioLegend, San Diego, CA) prior to staining with D^b^LT359 tetramers (NIH Tetramer Core Facility, Atlanta, GA) and antibodies to the following molecules: CD8α (53–6.7), CD44 (IM7), CD11b (M1/70), CD45(30-F11), CD69 (H1.2F3), CD103 (M290), IFN-γ (XMG1.2), and H-2D^b^ (KH95) purchased from BD Biosciences (San Diego, CA); and PD-1 (RMP1-30), PD-L1 (MIH5), Eomes (Dan11mag), MHC-II (M5/114.15.2), Tim3 (RMT3-23), 2B4 (eBio244F4), and Lag3 (eBioC9B7W) purchased from eBioscience; and CD4 (RM4-5), CD45 (30-F11), CD11b (M1/70), FoxP3 (MF-14), CD25 (3C7), T-bet (4B10), PD-L2 (TY25), CD11c (N418), and CD127 (A7R34) from Biolegend (San Diego, CA). Brain and spleen cells were stimulated with 1 μM LT359-368 peptide (SAVKNY(Abu)SKL), no peptide or peptide-pulsed IFN-γ-treated BMDCs for 5-6 h in the presence of brefeldin A, stained for viability and surface markers, fixed, and permeabilized with CytoFix/CytoPerm (BD Biosciences, San Diego, CA), then stained for intracellular IFN-γ. Anti-IFN-γ staining in the absence of peptide was <1% of CD8^+^ CD44^hi^ gated cells (data not shown). Samples were acquired on an LSR II or LSRFortessa (BD Biosciences, San Diego, CA) and data analyzed using FlowJo software (Tree Star, Ashland, OR).

### Intracerebroventricular (i.c.v.) Cannulation

Prior to surgery, ALZET osmotic pumps (Model 2002, DURECT Corporation, Cupertino, CA) were loaded with 6 mg/ml control rat IgG (Jackson ImmunoResearch, West Grove, PA) or PD-L1 rat IgG (Clone 10F.9G2; BioXCell, West Lebanon, NH), according to manufacturer's instructions, and connected to L-shaped cannulas (ALZET Brain Infusion Kit 3) with tubing trimmed to 2 cm. Pumps were incubated overnight at 37°C in autoclaved PBS to activate flow. Surgical procedures were performed similarly to those described (40). Osmotic pumps were implanted subcutaneously via a scalp incision. After removal of soft tissue from the skull, the cannula was positioned into the left lateral ventricle (1.0 mm lateral to midline, 0.1 mm posterior to bregma, and 3.0 mm dorsoventral to the skull) and secured with ALZET Loctite adhesive. The scalp was sutured over the cannula. i.c.v. antibody administration lasted 14 days at a flow rate of 12 μl/day. Delivery of antibody to the lateral ventricle was confirmed by cutting the brain at the site of cannulation and measuring the volume of antibody remaining in the pump.

### Micropipette Adhesion-Frequency Assay

CD8 T cells from brains were isolated using magnetic bead-based positive selection columns (Miltenyi, Bergisch Gladbach, Germany). Coating of human RBCs with the peptide-MHC (p-MHC) D^b^LT359 monomers, quantification of binding events, TCR surface densities, and TCR affinity calculations were performed as described earlier (27, 41, 42). An adhesion frequency ≥ 0.1 between a T cell and a D^b^LT359–coated RBC is scored as Ag-reactive.

### Luxol Fast Blue (LFB)-Periodic Acid Schiff (PAS)-Hematoxylin Histology

Mice were anesthetized with ketamine and xylazine, perfused transcardially with 10 mL of 10% heparin in PBS and 10 mL of 10% neutral buffered formalin (NBF). After perfusion, heads were removed and immersed in 10% NBF overnight at room temperature. The next day, brains were sectioned on a coronal brain cutting matrix. Formalin fixed-paraffin embedded (FFPE) samples were deparaffinized, then 10 μm sections stained with LFB-PAS, and counterstained with Harris-modified hematoxylin (Fisher), as described (43). LFB-PAS stained sections were digitally imaged on a Keyence BZ-X710 all-in-one fluorescence microscope (4x magnification) and stitched together using ImageJ software (National Institutes of Health, Bethesda, MD). Analysis of myelination in the white matter tracts was performed as described (44).

### Immunofluorescence Microscopy

Anesthetized mice were perfused transcardially with heparinized PBS followed by 10% NBF. Three-mm pieces were coronally using a cutting matrix and immersed overnight in 10% NBF and then embedded in paraffin. Ten μm FFPE brain sections were deparaffinized and rehydrated. Antigen retrieval was then performed using 10 mM sodium citrate buffer (pH 6.0). Brain sections were stained with anti-NeuN (Clone A60; Millipore, Darmstadt, Germany) or anti-APC (Clone CC-1; Abcam, Cambridge, UK) for 30 min at room temperature using the Mouse-On-Mouse Fluorescein Kit (Vector, Burlingame, CA), then stained overnight at 4°C with rabbit anti-VP1 [graciously provided by R. Garcea (University of Colorado, Boulder, CO)] followed by donkey anti-rabbit IgG conjugated to Alex Fluor 594 (Jackson ImmunoResearch, West Grove, PA). Astrocyte staining was performed using directly conjugated anti-GFAP (Clone GA5; eBioscience, San Diego, CA). Tissue sections were then mounted with ProLong Gold Anti-Fade Reagent with DAPI (Life Technologies, Carlsbad, CA). Images were acquired using a Leica DM4000 B LED microscope (Leica-Camera, Wetzlar, Germany).

### NanoString Gene Expression Analysis

RNA was isolated from a 2-mm thick brain section from the left hemisphere of the cerebrum using the Maxwell® 16 simplyRNA tissue kit with an in-solution DNAse digestion step. RNA from uninfected control groups (2 mice/pool) and infected groups (3 mice/pool) were subjected to NanoString gene expression analysis. 100 ng RNA was used to assess the expression of 254 mouse inflammatory genes provided in the nCounter® Inflammation mouse panels. Fold changes and *p*-value for the genes were calculated using nSolver software. Fold changes higher than 1.5 fold and *p* ≤ 0.05 were considered significant. The gene list was imported into the Ingenuity Pathway Analysis (IPA) tool (Qiagen, Redwood City, CA) for enrichment analysis of the pathways and upstream regulators, using Ingenuity Knowledge Base (IKB) as reference data and the contextual analysis settings for mouse tissues (Supplementary Table 1). The enrichment data and the *p*-values, calculated by Fisher's exact test, were exported and plotted using ggplot2 package in R software. Principal component analysis (PCA) was performed using START: Shiny Transcriptome Analysis Resource Tool hosted at http://kcvi.shinyapps.io/START (46).

### Statistical Analysis

*p*-values were determined by Mann Whitney, Wilcoxon matched-pairs signed rank test, or one-way or two-way ANOVA using GraphPad Prism software (La Jolla, CA). All *p* < 0.05 were considered significant.

## Results

### MuPyV-Infected Glial Cells and Infiltrating Monocytes Express High Levels of PD-L1

**Figure 1:**
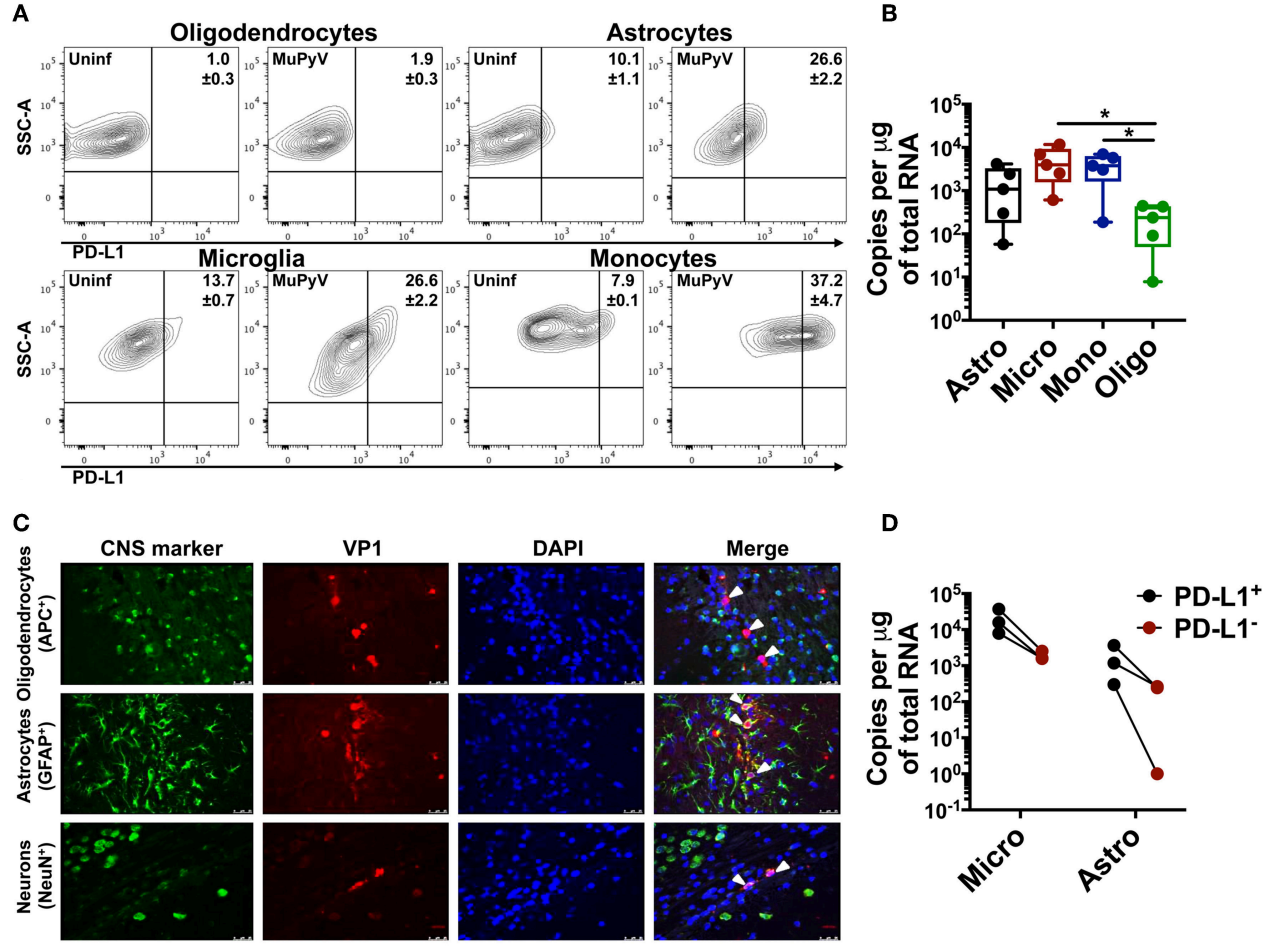
Neural cells express PD-L1. **(A)** Representative contour plots with frequency of PD-L1^+^ oligodendrocytes (CD11b^neg^/CD45^neg^/O4^+^), astrocytes (CD11b^neg^/CD45^neg^/GLAST^+^), microglia (CD11b^hi^/CD45^int^) and infiltrating monocytes (CD11b^hi^/CD45^hi^) from mock inoculated controls and MuPyV-infected mice at 8 dpi. The gates were drawn on the basis of the fluorescence minus one (FMO) controls. **(B)** LT-Ag mRNA copy number from FACS-purified astrocytes (Astro), microglia (Micro), infiltrating monocytes (Mono), and oligodendrocytes (Oligo). C_t_ values were normalized to the amount of total RNA taken for cDNA synthesis. Each point represents data from a pool of 3 mice. **(C)** Fluorescence photomicrographs of FFPE brain tissue sections from mice euthanized at 4 dpi stained with antibodies specific for the indicated CNS cell markers (green) and for MuPyV capsid protein VP1 (red). Nuclei were counterstained with DAPI (blue). White arrows in merged images indicate VP1^+^ cells (magnification 400X). **(D)** LT-Ag mRNA copy numbers from FACS-purified PD-L1^+^ and PD-L1^−^ microglia and astrocytes. C_t_ values were normalized with the C_t_ value of TBP mRNA for each cell type between the PD-L1^+^ and PD-L1^−^ samples. Each point connected by a line indicates cells from a pool of 3 mice. Data are cumulative from two independent experiments with 2–4 mice per group. Two-way ANOVA with Tukey multiple comparison test was performed. Values represent mean ± SD; *p ≤ 0.05.

Using adoptively transferred transgenic CD8 T cells expressing a MuPyV-specific TCR, we previously showed that brain-resident, but not splenic, antiviral CD8 T cells were PD-1^hi^ (28). Here, we examined the expression of PD-1 ligands by microglia, oligodendrocytes, and astrocytes, as well as by infiltrating monocytes in mice acutely infected with MuPyV (Supplementary Figure 1). With the exception of oligodendrocytes, all of these cell types variably upregulated PD-L1 after i.c. MuPyV inoculation, with the infiltrating monocytes having the highest frequency of PD-L1^+^ cells (Figure 1A). None of these cells showed expression of PD-L2 (data not shown). Although each of these cell populations was infected by MuPyV, microglia and infiltrating monocytes expressed at least a log higher LT-Ag transcripts than oligodendrocytes (Figure 1B). The marginally higher expression of VP1 transcripts in astrocytes vs. oligodendrocytes, while not achieving statistical significance, reinforces previous studies showing that JCPyV more efficiently infects astrocytes than oligodendrocytes in brains of mice engrafted with human glial progenitor cells (47). We further found that astrocytes, but not oligodendrocytes, express the viral capsid protein, VP1 (Figure 1C), a result in line with the human chimeric glial mouse-JCPyV infection model showing that astrocytes and not oligodendrocytes support productive infection (47, 48). In an interesting observation, we found that PD-L1^+^ astrocytes and microglia harbored a higher viral LT-Ag mRNA load as well (Figure 1D). These data show that resident and infiltrating CNS cell types that express PD-L1 are also infected with MuPyV with a positive association between PD-L1 expression and virus infection.

### Sustained PD-1 Expression by Antiviral CD8 T Cells During MuPyV Encephalitis

**Figure 2:**
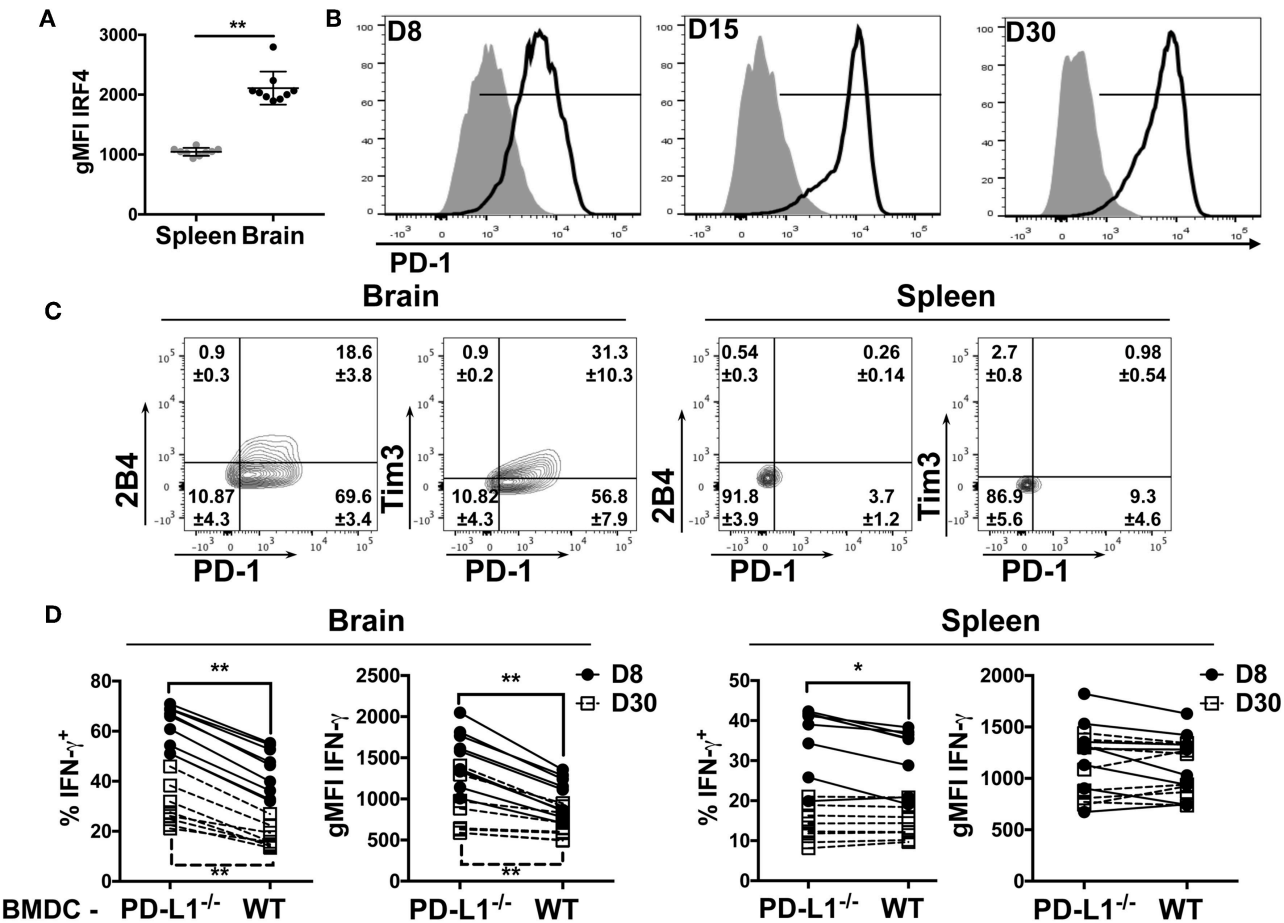
bT_RM_ express PD-1 during MuPyV infection. **(A)** Expression of IRF4 by D^b^LT359-specific CD8 T cells from brains and spleens of WT mice at 45 dpi in the indicated groups, represented as gMFI of the population. **(B)** Representative histograms of PD-1 expression by D^b^LT359^+^ CD8 T cells from brain (solid-line) or spleen (shaded) at the indicated dpi. **(C)** Representative contour plots with frequencies of Tim3/PD-1 and 2B4/PD-1 expressing brain-infiltrating virus-specific CD8 T cells at 45 dpi. **(D)** Frequency and gMFI of IFN-γ^+^ CD44^+^ CD8 T cells from the brain and spleen at the indicated timepoints upon *in vitro* stimulation with the LT359 peptide-pulsed BMDC from WT and PD-L1^−/−^ mice. Each point connected by a line indicates cells from one mouse. Data are from two independent experiments with 4-5 mice per group. Mann Whitney tests **(A)** between WT and PD-L1^−/−^ groups and Wilcoxon matched-pairs signed rank test **(D)** were performed. Values represent mean ± SD; *p ≤ 0.05, **p ≤ 0.01, not significant (ns) *p* > 0.05.

We reasoned that higher TCR affinity by the CD8 bT_RM_ would lead to augmented TCR signaling. Expression of the transcription factor IRF4 is reflective of TCR affinity and correlates with TCR signaling strength (49, 50). In confirmation of this prediction, we found that the CD8 bT_RM_ stained with tetramers for the dominant D^b^LT359 MuPyV epitope exhibited higher levels of TCR-signaling, as reflected by the higher expression of IRF4 in the brains than in spleens of mice at 45 days post-infection (dpi) (Figure 2A).

To understand if increased affinity of virus specific bT_RM_ may reflect selective accumulation of TCR clonotypes in the brain, we performed TCR repertoire analysis using the the TCR-β ImmunoSEQ assay. FACS-purified D^b^LT359 tetramer^+^ CD8 T cells were isolated from the brains and spleens of mice 30 dpi. Notably, ~75 of the top 100 TCR clones from the spleen were absent in the brain. Productive entropy, a measure of the diversity of the TCR sequences, was also higher for the D^b^LT359-specific memory CD8 T cells from the spleen than the D^b^LT359 specific bT_RM_ (Supplementary Figure 2A). The low diversity of TCR clones in the brain suggests that a subset of virus-specific peripheral CD8 T cells infiltrate or survive during the process of bT_RM_ development.

The dichotomy in PD-1 expression between brain and splenic MuPyV-specific TCR transgenic CD8 T cells was recapitulated by the endogenous D^b^LT359-specific CD8 T cell response. This dichotomy in PD-1 expression was recapitulated by the endogenous D^b^LT359-specific CD8 T cell response. D^b^LT359 tetramer^+^ CD8 T cells from the brain expressed PD-1 during the acute phase of infection (8 dpi), the expression peaked at 15 dpi, and was sustained into persistent infection (Figure 2B). In contrast, D^b^LT359-specific CD8 T cells in the spleen only transiently expressed a low level of PD-1 during acute infection (Figure 2B). A sizable fraction of PD-1^hi^ MuPyV-specific CD8 T cells in the brains of persistently infected mice also expressed the inhibitory receptors Tim3 and 2B4 (Figure 2C).

Next, we asked whether engagement of PD-1 by its ligand, PD-L1, functionally inhibited MuPyV-specific CD8 bT_RM_. Bone marrow-derived dendritic cells (BMDCs) from WT and PD-L1^−/−^ mice were treated with IFN-γ to maximize MHC class I and PD-L1 expression (Supplementary Figures 2B,C), then pulsed with LT359 peptide and used to stimulate T cells isolated from brains and spleens of MuPyV infected mice. LT359 peptide-stimulated CD8 T cells from brains had a higher frequency of IFN-γ^+^ cells and higher gMFI for IFN-γ at both 8 and 30 dpi when exposed to PD-L1^−/−^ BMDCs as compared to WT BMDCs. In contrast, D^b^LT359-specific CD8 T cells from the spleen showed only a modest increase in IFN-γ production at 8 dpi when stimulated by PD-L1^−/−^ BMDCs, a time point coincident with PD-1 expression (Figure 2D). Taken together, these data point toward PD-1-mediated inhibition of virus-specific CD8 T cell effector activity during MuPyV encephalitis.

### PD-L1 Deficiency Results in a Heightened Inflammatory Environment

**Figure 3:**
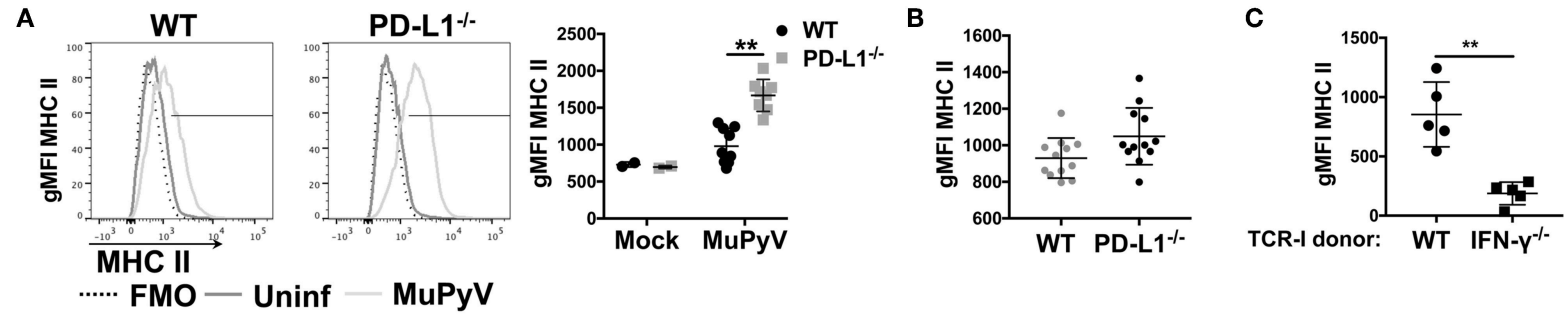
Lack of PD-1 signaling augments CD8 T cell IFN-γ production *in vivo*. **(A)** Representative histograms of MHC class II expression on microglia from WT and PD-L1^−/−^ mice at 8 dpi (left panel) with mean ± SD of MHC II gMFI (right panel). **(B)** MHC II gMFI (mean ± SD) on microglia at 45 dpi. **(C)** MHC II gMFI on microglia from WT mice at 8 dpi after i.v. transfer of IFN-γ sufficient or IFN-γ^−/−^ naïve TCR-I cells at day−1. Combined data from 2 to 3 independent experiments with 2–4 mice per group; each value indicates cells isolated from an individual mouse. Two-way ANOVA with Tukey multiple comparison test **(A)** or Mann Whitney test **(C)** was performed. **p ≤ 0.01.

Given that disruption of PD-1:PD-L1 signaling augmented IFN-γ production by anti-viral CD8 T cells *in vitro*, we hypothesized that PD-1 could be playing a role in controlling neuroinflammation *in vivo*. Upregulation of MHC class II expression on microglia is a commonly used indicator of neuroinflammation (9, 51, 52). Comparing MHC class II expression on microglia from brains of MuPyV infected WT and PD-L1^−/−^ mice, we found significantly higher I-A^b^ (MHC class II) surface expression on microglia from PD-L1^−/−^ mice at 8 dpi (Figure 3A); however, by 45 dpi MHC II expression levels on microglia of WT and PD-L1^−/−^ mice were comparable (Figure 3B). These data point toward an acute-to-persistent infection phase remission of the heightened inflammatory environment in the PD-L1^−/−^ mice. Furthermore, we found that brain microglia from mice that were adoptively transferred with IFN-γ-sufficient MuPyV-specific TCR transgenic CD8 T cells upregulated I-A^b^ expression, but no I-A^b^ upregulation was seen in mice that received IFN-γ deficient-transgenic TCR CD8 T cells (Figure 3C). These data support the likelihood that IFN-γ released by CNS-infiltrating, virus-specific CD8 T cells contributes to neuroinflammation during MuPyV encephalitis.

**Figure 4:**
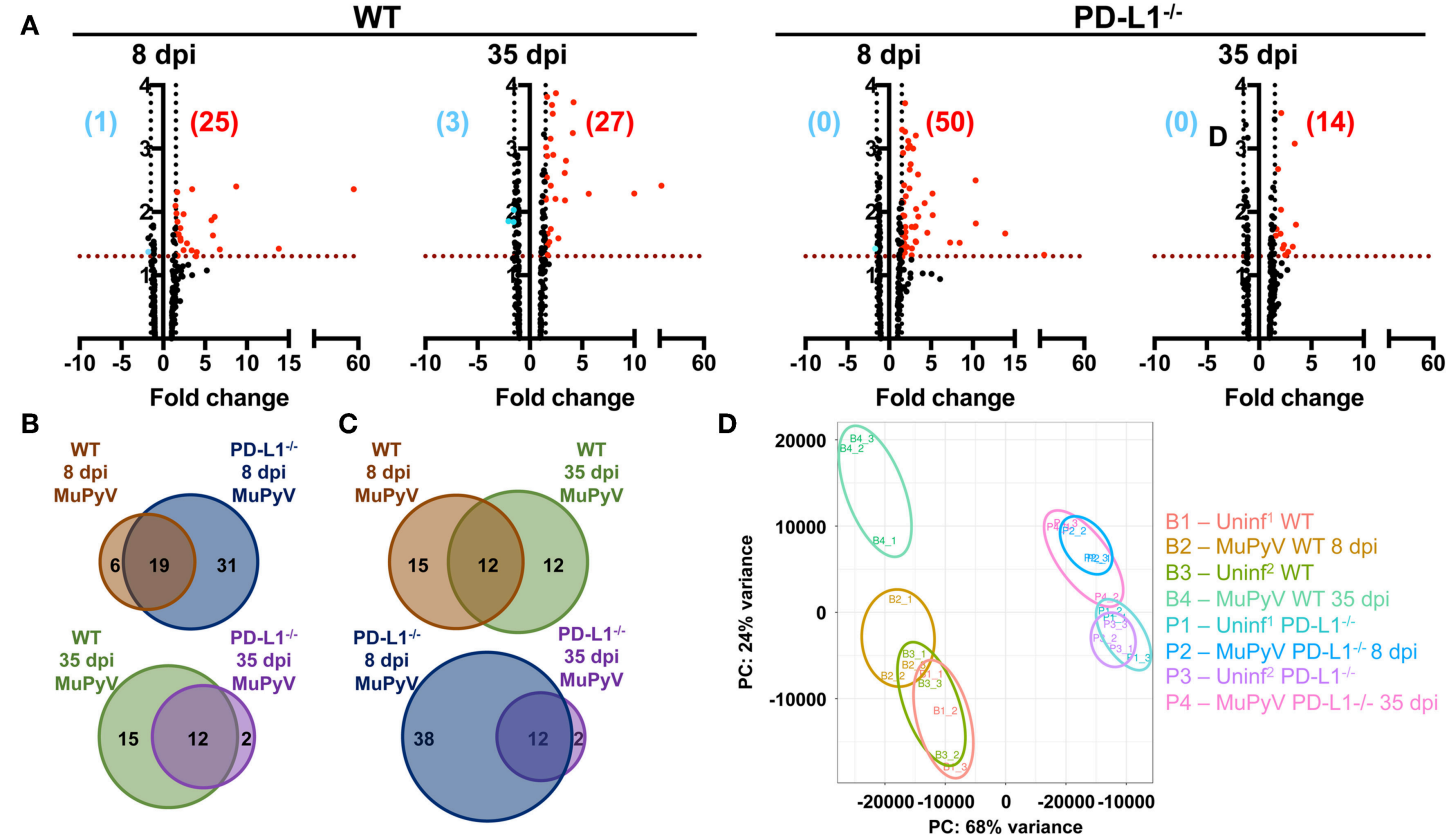
Brains of WT mice and PD-L1^−/−^ mice undergo dynamic and disparate changes in inflammatory transcriptomes from acute to persistent phases of infection. **(A)** Volcano plots representing differentially expressed genes (fold change ≥1.5 and *p* ≤ 0.05) of MuPyV infected WT brain samples vs. mock inoculated WT control brains and MuPyV infected PD-L1^−/−^ brains vs. mock inoculated PD-L1^−/−^ brains at 8 dpi and 35 dpi. Combined data from a total of 3 pools of 3 mice per pool. Downregulated and upregulated genes are represented in blue and red dots, respectively. Similarly, numbers in blue and red represent the number of downregulated and upregulated genes, respectively. The y-axis represents –log(*p-value*) for each gene in the NanoString mouse inflammation panel. **(B,C)** Venn diagrams showing the overlap in the differentially regulated genes between each group as indicated. **(D)** PCA of each pooled sample was performed using normalized counts of all genes in the array. Uninf^1^ and Uninf^2^ are two independent sets of mock infected controls samples run along with the 8 dpi and 35 dpi MuPyV-infected samples, respectively.

To obtain a more comprehensive view of the role of PD-1 signaling in controlling neuroinflammation, NanoString gene expression analysis using a 254 gene mouse inflammation panel was performed on brains from acutely (8 dpi) and persistently (35 dpi) infected mice. At 8 dpi, a higher number of genes were upregulated in PD-L1^−/−^ (50 genes) vs. WT mice (25 genes) (Figure 4A). However, in persistent infection, the inflammatory landscape profoundly changed, and did so differently between WT and PD-L1^−/−^ mice. For example, in WT mice, a number of genes were uniquely expressed in the 8 dpi and 35 dpi datasets. In contrast, the gene set upregulated in PD-L1^−/−^ mice at 35 dpi represented a subset of those same genes that were upregulated at 8 dpi (Figures 4B,C). These differences were further reinforced by principal component analysis (PCA) which showed that the inflammatory gene expression profile in the brains of WT mice at 35 dpi were significantly different from both uninfected as well as 8 dpi WT mice (Figure 4D). These observations suggest that the neuroinflammatory environment changes considerably over the course of MuPyV infection in the WT mice, while PD-L1^−/−^ mice show fewer differences between acute and persistent infection.

**Figure 5:**
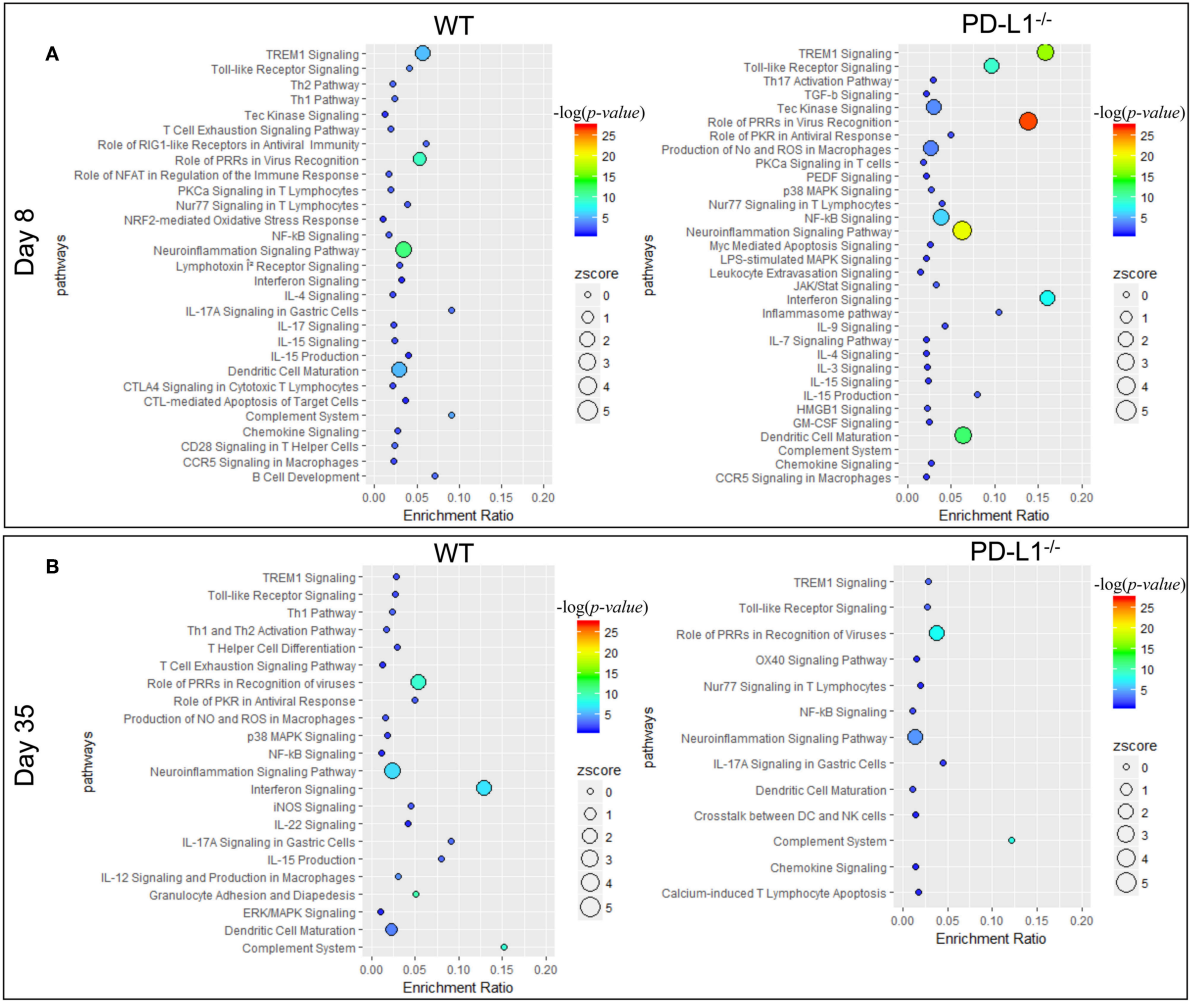
PD-1 signaling alters inflammatory pathways in the brain during MuPyV infection. Pathway enrichment analysis of the differentially expressed genes between MuPyV-infected WT vs. mock-inoculated WT control (left panel), and MuPyV-infected PD-L1^−/−^ vs. mock-inoculated PD-L1^−/−^ control brain (right panel) at 8 dpi **(A)** and 35 dpi **(B)** using IPA software. The y-axis represents pathway and the x-axis represents enrichment ratio. Bubble size represents the z-score and color represents the -log(*p-value*) calculated by Fisher's exact test.

Ingenuity pathway analysis (IPA) revealed major differences in the neuroinflammation signaling pathways in MuPyV-infected WT and PD-L1^−/−^ mice. A heightened inflammatory state is indicated in PD-L1^−/−^ mice by engagement of pathways involved in interferon signaling, dendritic cell maturation and recognition by Pattern Recognition Receptors (PRRs) at 8 dpi (Figure 5A). Conversely, at 35 dpi the neuroinflammation signaling pathway, interferon signaling, role of PRRs in virus recognition and iNOS signaling exhibited higher enrichment scores and –log(*p-value*) in WT mice than their PD-L1^−/−^ counterparts (Figure 5B). These data provide further evidence for a sustained inflammatory response in WT mice but not in PD-L1^−/−^ mice. Using upstream regulator analysis of IPA, we found that TNF-α and IFN-γ, mediator of CD8 T cell effector function and other inflammatory mediators like IL-15, IL-21, NOS2, STAT1, and NF-κB had higher z-score and –log(*p-value*) at 35 dpi in PD-L1^−/−^ mice than WT mice (Supplementary Figure 3). These data suggest that PD-1 protects against neuroinflammation during acute MuPyV infection, but paradoxically, ablation of this signaling pathways results in failure to sustain an inflammatory response during persistent infection.

### PD-L1-Deficiency Affects the T_RM_ Phenotype

**Figure 6:**
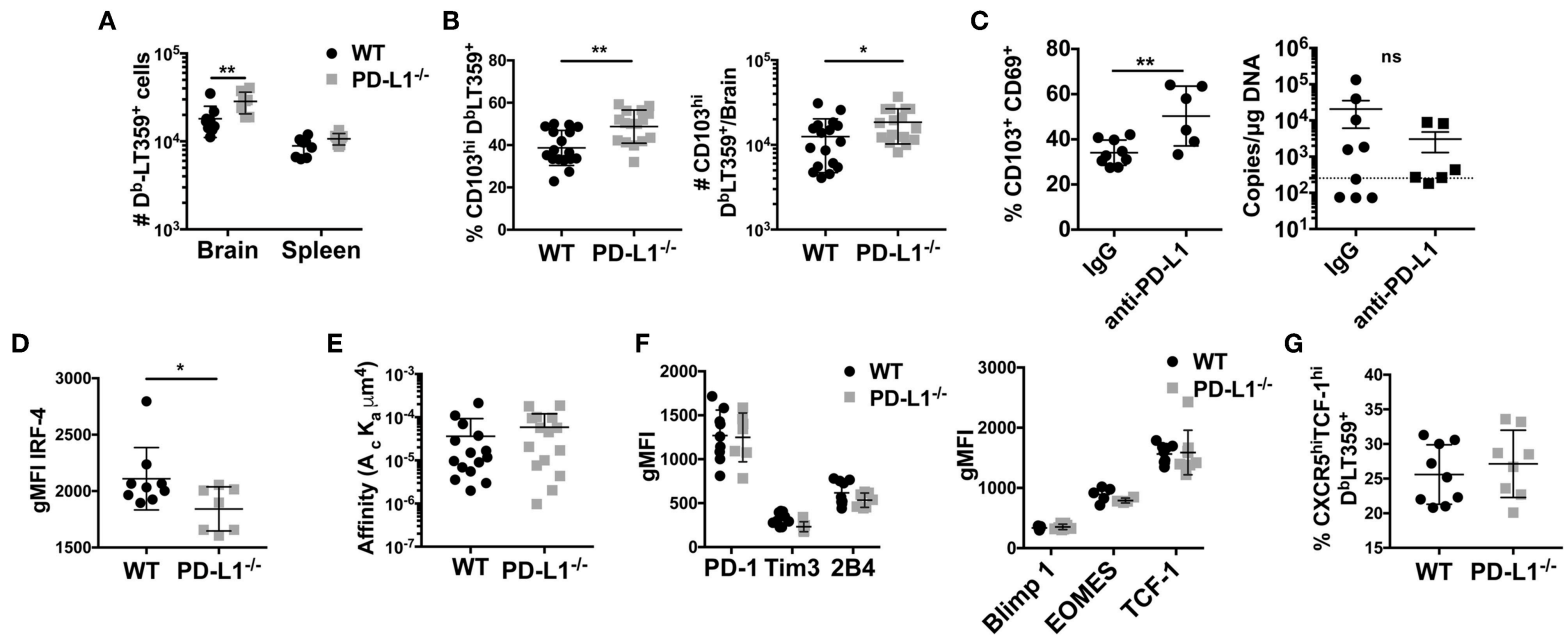
Lack of PD-1 signaling is associated with an increased frequency of CD103^+^ bT_RM_ **(A)** Number of D^b^LT359 specific CD8 T cells in WT and PD-L1^−/−^ mice. **(B)** Frequency and number of brain CD103^+^ D^b^LT359^+^ CD8 T cells in WT and PD-L1^−/−^ mice. **(C)** Frequency of CD103^+^D^b^LT359^+^CD69^+^ CD8 T cells and viral DNA load at 30 dpi in brains of WT mice with control IgG or PD-L1 antibody delivered by i.c.v. cannulae. **(D)** gMFI of IRF4 expression by D^b^LT359^+^ T cells at 9 and 45 dpi. **(E)** TCR affinities of D^b^LT359 monomer-binding CD8 T cells determined by 2D-micropipette adhesion assay. Data are from a pool of 5 mice. **(F)** gMFI of PD-1, Tim3, and 2B4 (left panel), and Blimp-1, EOMES, and TCF-1 (right panel). **(G)** Frequency of TCF-1^+^CXCR5^+^ D^b^LT359^+^ CD8 T cells from the brains of mice at 45 dpi. Data are from two to three independent experiments with 3–8 mice per group. Mann Whitney test between WT and PD-L1^−/−^ groups was performed in panels B, C, and D; two-way ANOVA with Tukey multiple comparison test in panel A was performed. Values represent mean ± SD; *p ≤ 0.05, **p ≤ 0.01, not significant (ns) *p*>0.05.

PD-1 signaling has been postulated to regulate CD8 T cell memory formation both in the context of peripheral as well as resident memory (10, 53, 54). In MCMV infection, PD-1 signaling promoted bT_RM_ formation, but had no effect on virus control (53). However, unlike in MCMV acute infection, PD-L1^−/−^ mice showed only a modest, albeit significant, increase in the number of the D^b^LT359-tetramer^+^ CD8 T cells; this increase was not seen in the spleen (Figure 6A). Unexpectedly, the frequency as well as number of D^b^LT359-specific CD8 T cells expressing CD103 were modestly higher in persistently infected PD-L1^−/−^ mice than WT mice (Figure 6B). We independently confirmed this apparent inverse relationship between PD-1 signaling and CD103 expression on MuPyV-specific CD8 T cells by continuous two-week delivery of anti-PD-1 vs. control IgG in persistently infected WT mice (Figure 6C). Interestingly, viral load was unaffected upon either *Pdcd1* ablation or antibody-mediated blockade of the PD-1 signaling (Figure 6C and Supplementary Figure 5C). TGF-β produced by CD25^+^ FoxP3^+^ CD4^+^ T cells induces CD103 (55). During the acute phase of infection, brain infiltrating CD25^+^ FoxP3^+^ CD4^+^ T cells were found to express PD-1. Also, PD-L1^−/−^ mice had moderately higher numbers of CD25^+^ FoxP3^+^ CD4 T cells compared to the WT mice (Supplementary Figures 4A,B). This increase coincides with higher TGF-β transcripts in the PD-L1^−/−^ mice (Supplementary Figure 4C). These data suggest that the expression of PD-1 on brain infiltrating CD4 T cells could be regulating the observed CD8 T cell response and phenotype in PD-L1^−/−^ mice.

Subcortical demyelination and enlargement of the ventricles are some of the hallmarks of JCPyV-associated-CNS-syndromes (31). To detect demyelination MuPyV encephalitis, LFB-PAS-hematoxylin staining was performed on vehicle i.c. injected and MuPyV infected brains. No change was seen at 4 dpi between control and infected WT mice (Supplementary Figure 5A). Small foci of demyelination and vacuolation were present in the cingulum bundle and the external capsule at 9 dpi, with enlarged ventricles and edema in the white matter tracts that became progressively worse by 30 dpi (Supplementary Figure 5A). Given the heightened neuroinflammation in the PD-L1^−/−^ mouse brains, we were surprised to see comparable levels of demyelination in persistently infected WT and PD-L1^−/−^ mice [19 dpi (Supplementary Figure 5B) and 30 dpi (data not shown)].

Because MuPyV-specific bT_RM_ express high affinity TCRs and are nearly all PD-1^hi^, we next investigated the possibility that PD-1 might influence the generation of these cells. At 8 dpi, there was no difference in expression of IRF4 by D^b^LT359-tetramer^+^ CD8 T cells between WT and PD-L1^−/−^ mice, but at 45 dpi the bT_RM_ from PD-L1^−/−^ mice showed lower IRF4 expression (Figure 6D). Decreased IRF4 expression was not only restricted to the immunodominant D^b^LT359 tetramer^+^ CD8 T cells, but was also seen in the D^b^LT359 tetramer^−^ CD8 T cells (Supplementary Figure 6A). To see if the difference in IRF4 expression stemmed from differences in TCR affinity, we performed 2D micropipette cell adhesion assays. Interestingly, no difference in TCR affinities between the D^b^LT359-specific CD8 T cells from WT and PD-L1^−/−^ mice was detected (Figure 6E). Expression of LT-Ag transcript in the brain was also found to be similar between WT and PD-L1^−/−^ mice, negating the possibility that differences in the expression of IRF4 could be ascribed to the differences in antigen expression (Supplementary Figure 6B). Thus, a mechanism other than modulation of TCR affinity comes into play to dampen TCR signaling strength by virus-specific CD8 T cells in the absence of PD-1 signaling.

In LCMV clone 13 chronic infection, circulating CD8 T cells undergo severe exhaustion in the absence of PD-1:PD-L1 signaling (10). Thus, we asked if MuPyV-specific CD8 bT_RM_ developing in the absence of PD-1 signaling undergo terminal exhaustion as an alternative explanation for lower TCR signal strength. Genetic deficiency of PD-L1, did not change the expression of PD-1, 2B4, or Tim3 by MuPyV-specific CD8 T cells (Figure 6F). Lack of PD-1 signaling also did not affect the expression of the transcription factors Eomes, Blimp-1 and TCF-1 (all known to be associated with exhaustion) by D^b^LT359-specific CD8 T cells (Figure 6F). TCF1^hi^CXCR5^hi^ CD8 T cells have been recently defined as the PD-1^+^ memory subset in LCMV clone 13 infections that exhibited less exhaustion and improved functionality upon PD-1 blockade (21, 22). In analogous fashion, we found that D^b^LT359-specific CD8 bT_RM_ in PD-L1^−/−^ mice had higher numbers of TCF1^hi^CXCR5^hi^ cells (Figure 6G). Taken together, these findings suggest that loss of PD-1 signaling during MuPyV-encephalitis led to increased CD103 expression and did not guide CD8 bT_RM_ toward terminal exhaustion.

### PD-L1 Deficiency Results in Impaired Virus Control Upon Re-infection

**Figure 7:**
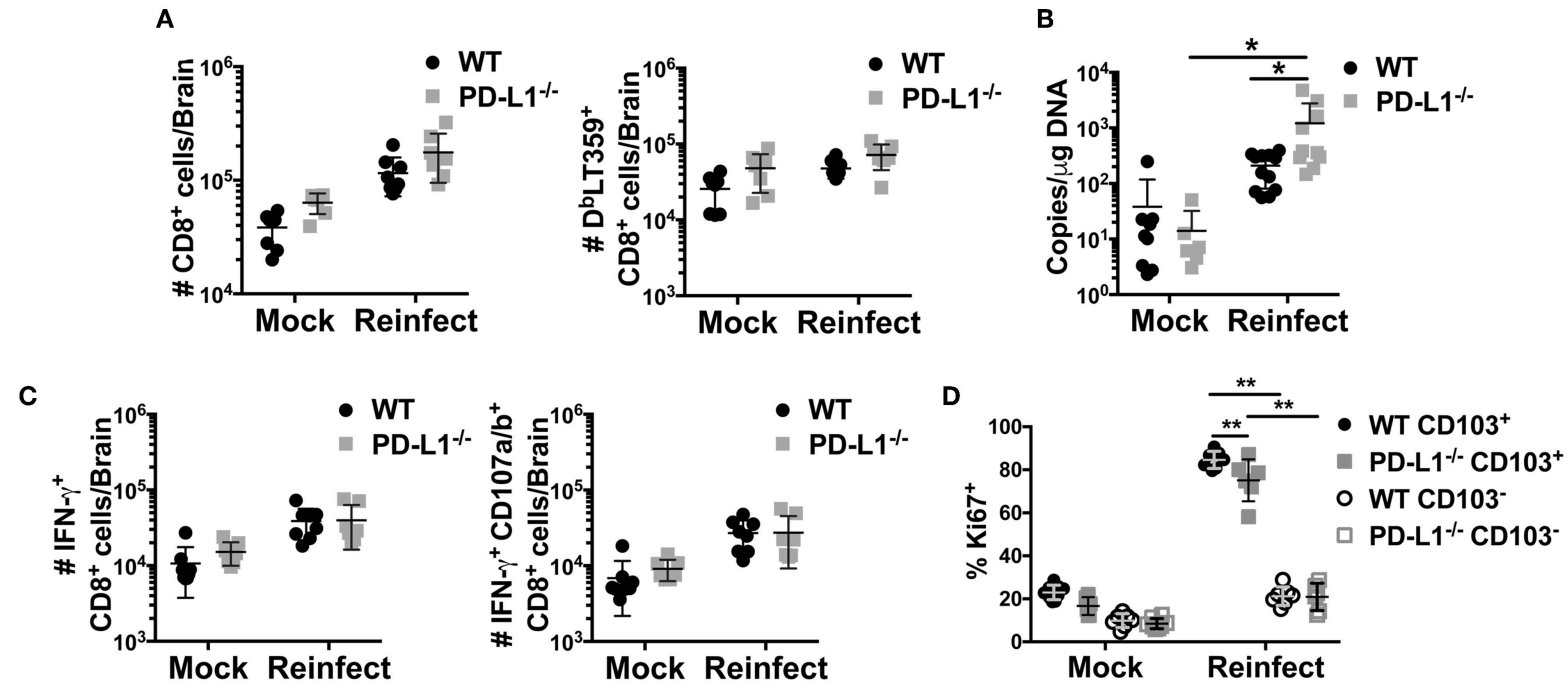
Lack of PD-1 signaling results in impaired viral control. WT or PD-L1^−/−^ mice were re-infected i.c. with MuPyV or mock inoculated at 35 dpi, then euthanized 5 days later. Total CD8 T cells and D^b^LT359^+^ CD8 T cells from brains were analyzed by flow cytometry. **(A)** Number of total CD8 and D^b^LT359^+^ CD8 T cells per brain, **(B)** Brain viral DNA genome copies by qPCR at 5 days after re-infection. **(C)** Number of IFN-γ^+^ (left panel) and IFN-γ^+^ CD107a/b^+^ (right panel) CD8 T cells upon *in vitro* stimulation with LT359 peptide. **(D)** Frequency of Ki67^+^ LT359^+^ CD103^+/−^ CD8 T cells upon reinfection. Data from two independent experiments with 3–5 mice per group. Two-way ANOVA with Tukey multiple comparison test was performed. Values represent mean ± SD; *p ≤ 0.05, **p ≤ 0.01. **(D)** Frequency of Ki67^+^ LT359^+^ CD103^+/−^ CD8 T cells upon reinfection.

Lack of PD-1 signaling due to genetic ablation of PD-L1 resulted in an increased fraction of CD103^+^ MuPyV-specific CD8 bT_RM_ but did not affect virus levels. CD103 expression has been associated with improved functionality in CD8 T_RM_ cells (56). To test bT_RM_ recall responsiveness, we re-inoculated >45 dpi WT and PD-L1^−/−^ mice i.c. with MuPyV and compared their ability to control this homologous virus challenge. Five days after re-inoculation, persistently infected WT and PD-L1^−/−^ mice showed similar increases in numbers of total CD8 T cells, but significantly more D^b^LT359-specific CD8 T cells were seen only in the brains of the re-infected WT mice (Figure 7A). The difference between total and virus-specific CD8 T cell numbers in the re-infected WT mice suggested that this increase was predominantly due to recall expansion of anti-MuPyV CD8 bT_RM_ rather than recruitment. Importantly, re-infected PD-L1^−/−^ mice had an ~87-fold increase in virus load in the brain, whereas WT mice showed roughly a 6-fold increase in virus levels (Figure 7B). D^b^LT359-specific CD8 T cells from the brains of WT and PD-L1^−/−^ mice at day 5 post-challenge also exhibited similar abilities to produce IFN-γ and degranulate (e.g., CD107a/b^+^) upon LT359 peptide stimulation (Figure 7C). The recall response upon re-infection was mainly due to the activation of CD103^+^ cells, as only CD103^+^ cells showed high proliferative potential as indicated by their Ki67 expression. We also observed a moderate decrease in Ki67^+^ cells in PD-L1^−/−^ mice (Figure 7D). These observations are in line with data in Figure 5B and Supplementary Figure 3, showing PD-1 signaling deficiency results in failure to sustain an inflammatory environment, resulting in poorer virus control. Moreover, these data support the likelihood that neuroinflammatory factors induced by PD-1 signaling are required to maintain immunologic defense against resurgence of a persistent viral encephalitis.

## Discussion

Accumulating evidence indicates that expression of PD-1 and CD103 by CD8 bT_RM_ is dependent on virus context. For example, VSV and LCMV-Armstrong generate PD-1^−^ CD103^+^ CD8 bT_RM_, but CD8 bT_RM_ in TMEV and MCMV encephalitis are PD-1^+^ CD103^+^ (53, 57–59). In contrast, during persistent MuPyV encephalitis, virus-specific CD8 T cells are uniformly PD-1^+^, but only ~40% express CD103 (28). In addition, irrespective of CD103 status, MuPyV-specific CD8 bT_RM_ are maintained stably independent of resupply from the circulation; and CD103^+^ and CD103^−^ cells have highly overlapping transcriptomes (28, 60). However, the CD103^+^ subset preferentially produces IFN-γ (28, 60) and proliferates upon i.c. MuPyV re-infection (Figure 7D). Moreover, PD-L1^−/−^ mice have diminished ability to control virus levels upon homologous virus re-infection, despite having a higher proportion of CD103^+^ MuPyV-specific CD8 bT_RM_ than WT mice. Although CD103 expression has been linked to CD8 bT_RM_ formation in response to VSV CNS infection (61), other studies show that CD8 T cell motility in the small intestine mucosa and brain are not dependent on CD103 (58, 62). In addition, intestinal pathogen-specific CD8 T_RM_ to oral *Yersinia pseudotuberculosis* infection are comprised of CD103^+^ and CD103^−^ populations, with the latter preferentially localizing to infectious foci (63). In sum, the potential contributions of CD103 to anatomical localization and function of CD8 T cells in different tissues and in the setting of different pathogen infections remain to be determined.

CD4 T cell-derived cytokines may be involved in upregulating CD103 on CD8 T_RM_. Nearly all MuPyV-specific CD8 T cells in the brains of CD4 T cell-deficient mice are CD103^−^ (60). TGF-β induces CD103 on T_RM_ (64–66); more TGF-β1 transcripts are detected in brains of PD-L1^−/−^ than WT mice persistently infected with MuPyV (Supplementary Figure 4C). In this connection, all CD25^+^ CD4 T cells isolated from the brains of acutely MuPyV infected mice are PD-1^+^ and a fraction are FoxP3^+^ as well (Supplementary Figures 4A,B). Together, these data raise the possibility that, in the absence of inhibitory PD-1 signaling, CD4 T cells in the brain produce more cytokines that induce CD103 expression on CD8 bT_RM_.

PD-1 expression by CD8 T cells may not inevitably result in T cell exhaustion. PD-1 has been shown to be dispensable for CD8 T cell exhaustion in chronic LCMV infection (10). Severity and duration of chronic viral infections are associated with expression of multiple inhibitory receptors by CD8 T cells in addition to PD-1 (67). In infected WT and PD-L1^−/−^ mice, however, PD-1^+^ D^b^LT359 specific CD8 bT_RM_ cells expressed equivalent levels of Tim3 and 2B4. Expression of the Blimp1, Eomes and TCF-1 transcription factors was also unaffected by the absence of PD-1 signaling. LT359 peptide-stimulated bT_RM_ from WT and PD-L1^−/−^ mice were similarly capable of IFN-γ production and degranulation, indicating retention of function independent of PD-1 signaling. MuPyV-specific CD8 bT_RM_ from WT and PD-L1^−/−^ mice also expressed similar levels of PD-1 during persistent infection. We recently reported that PD-1 downregulation on splenic MuPyV-specific CD8 T cells is followed by *Pdcd1* promoter loci remethylation (28). PD-1 is stably expressed on anti-MuPyV bT_RM_ in PD-L1^−/−^ mice. These data support the interpretation that PD-1 signaling does not regulate the methylation status of the *Pdcd1* gene promotor. In the brains of PD-L1^−/−^ mice a number of upstream regulators were enriched during acute infection but reduced or below threshold during persistent infection (e.g., IFN-γ, IL-15, NOS, NFATC2, and IL12-A), suggesting dynamic epigenetic changes in these genes in the absence of PD-1 signaling.

Microglia, astrocytes and oligodendrocytes express PD-L1, under various inflammatory conditions, such as those induced by MCMV and TMEV CNS infections (8, 33, 68, 69). Yet, PD-L1 in mouse coronavirus encephalomyelitis is only transiently and poorly expressed on microglia, although oligodendrocytes exhibit high and sustained expression (8). In MCMV encephalitis, microglia and astrocytes express PD-L1 (9). In MuPyV infection, PD-L1 was expressed on the infiltrating monocytes, microglia and astrocytes, but not on oligodendrocytes. IFN-γ is a potent inducer of PD-L1 (9). The positive correlation between viral LT-Ag mRNA and PD-L1 expression in MuPyV-infected brain cells could be a result of contact of the infected cells with IFN-γ-secreting CD8 T cells. A similar mechanism termed “adaptive resistance” dampens anti-tumor CD8 effector activity (70, 71). The alternate possibility that MuPyV-infected cells upregulate PD-L1 as a virus-immune evasion strategy is less likely, given that oligodendrocytes, which are also infected, fail to express PD-L1.

Using a NanoString mouse inflammatory gene expression array, we uncovered a PD-L1-dependent difference in the inflammation transcription landscape over the course of MuPyV infection. Compared to WT mice, PD-L1 deficiency during acute infection involved upregulated expression of more genes and enrichment of neuroinflammatory pathways (Figures 4, 5 and Supplementary Figure 3). Of these, brains of PD-L1^−/−^ mice showed exclusive enrichment of NF-κB and apoptotic pathways, as well as IL-9, IL-12, and IL-3 signaling pathways (Figure 5 and Supplementary Figure 3). Although the inflammation transcriptome changed considerably as infection transitioned from acute to persistent infection in WT mice, it failed to do so in PD-L1^−/−^ mice (Figure 4D). Despite this difference, WT and PD-L1^−/−^ mice shared the IL-15 signaling pathway as an upstream regulator. IL-15 is of particular interest, because it is expressed by CNS parenchymal cells as well as by infiltrating immune cells in response to an ongoing inflammatory insult (72). In addition, IL-15 limits apoptosis in neuronal cells and suppresses nitric oxide production in neurons (72). The importance of IL-15 in memory CD8 T cell differentiation and establishing CD8 T_RM_ in barrier tissues are well documented (73–76). An intriguing possibility is that a deficiency in IL-15 in PD-L1 deficient mice underlies the compromised ability of CD8 T cells to control virus levels upon re-infection and promotes neural cell death during persistent infection.

The interferon signaling pathway also showed pronounced differences between WT and PD-L1^−/−^ mice. Genes in this pathway were found to be significantly enriched in persistently, but not acutely infected, WT mice with the reverse seen in PD-L1^−/−^ mice (Figure 5). Type I and II IFNs have been shown to inhibit JCPyV replication in glial cells *in vitro* (77, 78). In a clinical trial to evaluate IFN-γ as a prophylactic therapy for opportunistic infections in HIV^+^ patients, none receiving IFN-γ developed PML while 10% of those in the placebo group did (79). Type I IFNs have been shown to enhance the cytolytic activity of airway-resident memory CD8 T cells a secondary respiratory virus challenge (80). We reported that Type I and Type II IFNs inhibit MuPyV replication *in vitro* and in peripherally inoculated mice, that MuPyV infection *in vitro* and *in vivo* induces IFN-β mRNA, and that Type I IFN regulates MuPyV-specific CD8 T cell memory differentiation and function (81, 82). JCPyV, as well as BKPyV and SV40, infections induce a STAT1-dependent upregulation of interferon-stimulated genes and production of IFN-β (83, 84). Thus, differences in IFN signaling pathways in WT and PD-L1^−/−^ mice may reflect dynamic changes over the course of MuPyV encephalitis in Type I IFN-mediated innate immunity and/or Type II IFN production by CNS-infiltrating virus-specific T cells. It merits emphasizing, however, that the NanoString data do not establish a temporal link between PD-1 signaling and CD8 bT_RM_ development.

In summary, PD-1 signaling plays a critical role in regulating the immune response against MuPyV infection of the CNS. Defects in the PD-1:PD-L1 signaling pathway may lead to increased neuroinflammation at the peak of the CD8 bT_RM_ response, but neuroinflammation was not maintained during the persistent phase of this viral encephalitis. Thus, the inhibitory functions of PD-1 are necessary for the generation of a controlled, sustained inflammatory response in the CNS. These findings offer insights into the role of PD-1 in modulating an immune response of a persistent viral infection in the CNS beyond its documented function as a CD8 T cell inhibitory receptor.

## Ethics Statement

Institutional Animal Care and Use Committees and the Department of Comparative Medicine at the Pennsylvania State University College of Medicine. The Pennsylvania State University College of Medicine Animal Resource Program is accredited by the Association for Assessment and Accreditation of Laboratory Animal Care International (AAALAC). The Pennsylvania State University College of Medicine has an Animal Welfare Assurance on file with the National Institutes of Health's Office of Laboratory Animal Welfare; the Assurance Number is A3045-01.

## Author Contributions

Shwetank, EF, and AL contributed to the conception and design of the study and wrote the manuscript. Shwetank, EF, TM, HR, MT, ML, CN-W, GJ, and JC performed experiments, analyzed data, and performed the statistical analysis. All authors contributed to manuscript revision, read and approved the submitted version.

### Conflict of Interest Statement

The authors declare that the research was conducted in the absence of any commercial or financial relationships that could be construed as a potential conflict of interest.
