# Supplementary material for: PD-1 Dynamically Regulates Inflammation and Development of Brain-Resident Memory CD8 T Cells During Persistent Viral Encephalitis
Source: Front Immunol. 2019 Apr 17;10:783. doi: 10.3389/fimmu.2019.00783 (PMC6499176; doi:10.3389/fimmu.2019.00783)
Supplement: Supplementary file 1 [file Table_1.docx]

**Supplementary Table1: List of differentially expressed genes**

| **8 dpi WT. MuPyV vs WT mock control** | | |
| --- | --- | --- |
| Probe Name | Fold change | *p value* |
| Ccl5 | 55.86 | 0.00436633 |
| Cxcl10 | 13.8 | 0.03799578 |
| H2-Eb1 | 8.72 | 0.0039488 |
| Cxcl9 | 6.77 | 0.03891008 |
| Ccl2 | 6.14 | 0.0119931 |
| Ifi27l2a | 5.93 | 0.02350074 |
| Ccr2 | 5.75 | 0.01358807 |
| Cfb | 4.02 | 0.04319385 |
| Irf7 | 3.91 | 0.05012091 |
| Ccl8 | 3.46 | 0.00438785 |
| Oas1a | 3.39 | 0.04033577 |
| Ccl7 | 2.83 | 0.03120551 |
| Tlr8 | 2.44 | 0.01079896 |
| C4a | 2.43 | 0.04000231 |
| Ltb | 2.08 | 0.02844019 |
| Ccl19 | 2.06 | 0.02587602 |
| Maff | 2.05 | 0.01796745 |
| Fasl | 1.83 | 0.02248792 |
| Tlr1 | 1.71 | 0.04927757 |
| Ccl22 | 1.7 | 0.01424015 |
| Cd86 | 1.67 | 0.0048842 |
| Tlr9 | 1.61 | 0.04812906 |
| Ccr7 | 1.59 | 0.01051588 |
| C1ra | 1.54 | 0.02213169 |
| **35 dpi WT. MuPyV vs WT mock control** | | |
| Probe Name | Fold change | *p value* |
| Ccl21a | 21.65 | 0.00385135 |
| Ccl5 | 9.98 | 0.00514917 |
| C3 | 5.63 | 0.00519691 |
| Ifi27l2a | 4.18 | 0.00018602 |
| Iigp1 | 4.11 | 0.00057212 |
| C4a | 3.45 | 0.00155068 |
| Cxcl10 | 3.36 | 0.00660575 |
| H2-Eb1 | 3.34 | 0.00243282 |
| Cxcl9 | 2.73 | 0.02621192 |
| Ifi44 | 2.5 | 0.00013401 |
| Ccr2 | 2.44 | 0.00623159 |
| Oas1a | 2.24 | 0.00125597 |
| Tlr1 | 2.19 | 0.00028228 |
| Ifit3 | 2.13 | 0.00020535 |
| Irf7 | 2 | 0.00386486 |
| Ccl17 | 2 | 0.01870517 |
| Ifit1 | 1.99 | 0.00070004 |
| Ccl2 | 1.86 | 0.0298048 |
| Tlr2 | 1.7 | 0.03356503 |
| C3ar1 | 1.69 | 0.02199041 |
| C7 | 1.69 | 0.04785694 |
| Ccl8 | 1.66 | 0.00015249 |
| Mx2 | 1.64 | 0.00131046 |
| C1s | 1.62 | 0.00285835 |
| Stat1 | 1.56 | 0.00095156 |
| Ptger3 | 1.54 | 0.00639239 |
| Irf1 | 1.52 | 0.00605169 |
| **8 dpi PD-L1^-/-^. MuPyV vs PD-L1^-/-^ mock control** | | |
| Probe Name | Fold change | *p value* |
| Ccl5 | 23.76 | 0.04736375 |
| Ccr2 | 16.08 | 0.00110798 |
| H2-Eb1 | 13.87 | 0.02187588 |
| Ifi27l2a | 10.35 | 0.01525928 |
| Cfb | 10.32 | 0.00318571 |
| C3 | 8.43 | 0.03062701 |
| Iigp1 | 7.28 | 0.03035948 |
| Irf7 | 5.22 | 0.0112353 |
| Ltb | 5.19 | 0.00514403 |
| C4a | 4.57 | 0.02130483 |
| Oas1a | 4.24 | 0.00722886 |
| Stat1 | 3.49 | 0.01187763 |
| Itgb2 | 3.46 | 0.00255816 |
| Oasl1 | 3.21 | 0.00902696 |
| Ifi44 | 3.21 | 0.02980675 |
| Tlr1 | 3.2 | 0.0006245 |
| Oas2 | 3.16 | 0.01697441 |
| Ccl8 | 3.16 | 0.02898082 |
| Irf1 | 3.04 | 0.01735609 |
| Fasl | 2.86 | 0.00100066 |
| Ifit3 | 2.72 | 0.0376232 |
| Tlr2 | 2.7 | 0.04843436 |
| Tlr9 | 2.69 | 0.01727043 |
| C3ar1 | 2.58 | 0.00176794 |
| Ccl19 | 2.46 | 0.00214508 |
| Tlr8 | 2.45 | 0.00089403 |
| Cd86 | 2.42 | 0.00426473 |
| C1qb | 2.29 | 0.00075515 |
| C1qa | 2.26 | 0.0009792 |
| C2 | 2.12 | 0.03566774 |
| Irf5 | 1.98 | 0.00565792 |
| Ptger4 | 1.97 | 0.02069694 |
| C1s | 1.94 | 0.01726598 |
| Cxcr4 | 1.92 | 0.02523151 |
| Chi3l3 | 1.92 | 0.03636526 |
| Tlr7 | 1.91 | 0.00019205 |
| Tyrobp | 1.9 | 0.00053799 |
| Ccr7 | 1.88 | 0.04397973 |
| Ccr1 | 1.8 | 0.01148603 |
| Trem2 | 1.78 | 0.00377522 |
| C1ra | 1.77 | 0.03122942 |
| Nlrp3 | 1.69 | 0.00705114 |
| Maff | 1.68 | 0.00117375 |
| Tlr4 | 1.68 | 0.01134686 |
| Ifit2 | 1.58 | 0.05096475 |
| Tgfb1 | 1.56 | 0.00050248 |
| Stat2 | 1.56 | 0.02202979 |
| Tlr6 | 1.55 | 0.04754685 |
| Il6ra | 1.53 | 0.00703157 |
| Nod1 | 1.53 | 0.03797203 |
| **35 dpi PD-L1^-/-^. MuPyV vs PD-L1^-/-^ mock control** | | |
| Probe Name | Fold change | *p value* |
| C3 | 3.53 | 0.01592232 |
| C4a | 3.4 | 0.00083654 |
| Ccl5 | 3.23 | 0.03531108 |
| H2-Eb1 | 2.77 | 0.04273495 |
| Ccl17 | 2.49 | 0.04778368 |
| Ifi27l2a | 2.37 | 0.03323461 |
| Ccl8 | 2.25 | 0.0375387 |
| C3ar1 | 2.12 | 0.00027455 |
| Tlr2 | 2.09 | 0.00925968 |
| Ccr2 | 2.04 | 0.0221377 |
| C1s | 1.81 | 0.00210778 |
| Tlr6 | 1.68 | 0.01867157 |
| Ifi44 | 1.61 | 0.02367276 |
