# Supplementary material for: PD-1 Dynamically Regulates Inflammation and Development of Brain-Resident Memory CD8 T Cells During Persistent Viral Encephalitis
Source: Front Immunol. 2019 Apr 17;10:783. doi: 10.3389/fimmu.2019.00783 (PMC6499176; doi:10.3389/fimmu.2019.00783)
Supplement: Supplementary file 2 [file Data_Sheet_1.docx]

**Supplementary Figure 1**. **Flow cytometry gating strategy for mouse neuronal cells.**

**Supplementary Figure 2. bT_RM_ TCR repertoire is less diverse than the splenic memory TCR repertoire, and BMDCs treated with IFN-γ induce MHC II and PD-L1 surface expression.** (A) ImmunoSeq TCR-β chain analysis of FACS-purified D^b^LT359 tetramer^+^ CD8 T cells from brains and spleens of wild type mice 45 days after MuPyV i.c. inoculation. Upper panel, representative productive frequency of the top 100 clones from spleen and brain in one of the five mice; lower panel, productive entropy of the TCR-β chain repertoire from five mice. (B) Frequency of the CD11c^+^CD11b^+^ cells in BMDC cultures. (C) MHC I, MHC II and PD-L1expression increases after overnight IFN-γ stimulation. A Wilcoxon matched-pair rank *t*-test was used to determine statistical significance. Cumulative data from five biological replicates. * *p*≤0.05.

**Supplementary Figure 3**. **Upstream regulator analysis using IPA of the NanoString data.** Red arrows mark the upstream regulators of interest.

**Supplementary Figure 4. CD4^+^CD25^+^FoxP3^+^ cell numbers and TGF-β1 expression levels are increased in brains of PD-L1^-/-^ mice.** (A) Gating strategy of CD4^+^CD25^+^FoxP3^+^ cells. PD-1 gMFI mean + SD (n = 5 mice) of the CD25^+^FoxP3^-^ and CD25^+^FoxP3^-^ CD4^+^ cells populations from brains of WT mice at 35 dpi. (B) Total number of CD4^+^CD25^+^FoxP3^+^ cells per brain. (C) mRNA counts of the TGF-β1 gene from given samples and conditions at 8 dpi, estimated in NanoString assay. Mann Whitney test (A) or two-way ANOVA with Tukey multiple comparison test were performed to calculate statistical significance. Values are mean ± SD; * *p*≤0.05, ** *p*≤0.01.

**Supplementary Figure 5. Demyelination of white matter brain tracts in MuPyV infected mice.** (A, B) Photomicrographs of Luxol fast blue-periodic acid Schiff-hematoxylin stained formalin-fixed paraffin-embedded (FFPE) brain sections from mice at indicated timepoints post-infection. The arrow in the top left panel indicates the i.c. infection needle track. The asterisk in the bottom right panel indicates a cutting artifact. Sections imaged were cut at approximately bregma +0.2 mm (top rows) and bregma -2 to -2.7 mm (bottom rows). Scale bars indicate 1 mm. (B) Representative photomicrographs showing myelination from bregma +0.2 mm region (left panel), and percent myelination of the white matter tracks in the brain (right panel) in WT and PD-L1-/- mice 19 dpi. (C) MuPyV genome copies determined by qPCR from genomic DNA isolated from brains and spleens at 30 dpi. All data are cumulative of 2-4 experiments, with 2-5 mice/group. Values are mean ± SD.

**A B**

**Supplementary Figure 6. IRF4 expression by D^b^LT359 tetramer-negative CD8 bT_RM_ and MuPyV LT-Ag transcripts in brains of persistently infected WT and PD-L1^-/-^ mice.** (A) IRF4 gMFI by D^b^LT359 tetramer^-^ CD8 T cells at 45 dpi. Symbols represent IRF4 expression levels by cells from individual mice. (B) Copies of LT-Ag transcripts in brains of WT and PD-L1^-/-^ mice 45 dpi. Mann Whitney test was performed to calculate statistical significance. Values are mean ± SD; ** *p*≤0.01.
